# Extending Metabolomic Studies of *Apis mellifera* Venom: LC-MS-Based Targeted Analysis of Organic Acids

**DOI:** 10.3390/toxins12010014

**Published:** 2019-12-28

**Authors:** Magdalena Pawlak, Agnieszka Klupczynska, Zenon J Kokot, Jan Matysiak

**Affiliations:** Department of Inorganic and Analytical Chemistry, Poznan University of Medical Sciences, Grunwaldzka 6 St, PL-60780 Poznan, Poland; magpawlak@ump.edu.pl (M.P.); aklupczynska@ump.edu.pl (A.K.); zkokot@ump.edu.pl (Z.J.K.)

**Keywords:** honeybee, mass spectrometry, venomics

## Abstract

Organic acids are important active small molecules present in venoms and toxins, which have not been fully explored yet. The aim of the study was the determination of organic acids in honeybee venom (HBV) samples by using liquid chromatography-tandem mass spectrometry (LC-MS/MS). Two protocols for sample preparation were employed. A solid-phase extraction was used for the determination of malonic acid, fumaric acid, glutaric acid, and kynurenic acid. A dilute-and-shoot method was optimal for: citric acid, malic acid, and succinic acid. Chromatographic separation was performed using a Synergi Hydro-RP column. Detection was performed on a triple-quadrupole mass spectrometer operating in multiple reaction monitoring mode. Among the analytes, glutaric acid and kynurenic acid were present in HBV samples in the lowest concentrations, whereas citric acid was the most abundant acid in each sample, and accounted for an average of 86 mg/g (8.6%) of the venom dry weight. Organic acids were discussed in terms of function. This is the first study in the available literature that provides specific data on the content of organic acids in HBV using a validated quantitative method.

## 1. Introduction

Venoms and toxins are one of the tools that ensure survival in the animal world. *Hymenoptera* is an order of insects comprising many venomous species. *Hymenopteran* sting triggers a systemic allergic reaction for prey or predator and can be deadly for the human organism causing anaphylactic shock. A honeybee (*Apis mellifera*) is a representative of *Hymenoptera* occurring almost all over the world [1]. Honeybee venom (HBV) is not only a danger for human when stung, but also has therapeutic properties. Nowadays, it is a subject of many studies due to its pharmacological and biological activities. Therefore, there are several medicinal applications of HBV into the human body for the treatment of some diseases to include Parkinson’s disease [2], multiple sclerosis [3], cancer [4], liver fibrosis [5], skin diseases [6], and pain [7] treatment. The second means of application of HBV is venom immunotherapy, which is designed to reduce the risk of a systemic reaction in the case of *Hymenoptera* stings [8]. Therefore, the cognition and standardization of HBV are necessary.

HBV is produced in specialized glands as a tool to defend a colony against predators [9]. It consists of many bioactive molecules such as peptides (i.e., melittin, apamin, adolapin), enzymes (i.e., phospholipase A_2,_ hyaluronidase, phosphatase), biogenic amines (i.e., histamine, epinephrine), and other nonpeptide compounds like amino acids or sugars [10,11]. Melittin makes up 50% of the dry weight of venom and triggers the toxicity of the venom. It causes pain, inflammation, and itching in high doses. However, it also has anti-inflammatory, anti-arthritic, and radiation protective effects [4,12,13,14]. From the enzymatic part of the venom, phospholipase A_2_ accounts for approximately 10%-12% of dry bee venom. Phospholipase A_2_ is the strongest allergen in HBV but its anti-tumor effect can also be well-known [4,15]. Nonpeptide compounds are a minority of dry HBV, however they can also be allergens and help in communication in a bee colony [4].

There are a lot of previous studies about the presence of peptides and enzymes in HBV [16,17,18] but there are very few papers on the content of low-molecular-weight compounds. Otherwise, in the available literature regarding the content of small molecules in animal venoms, authors rely on old papers, so there is a lack of source information and current research that could confirm the found data. The analysis of HBV on small molecules is possible due to modern analytical techniques. Development of “omic” technologies (proteomics, transcriptomics, genomics, and metabolomics) has revolutionized the study of venoms as they enable large-scale data collection and analysis. Two strategies can be employed in omics investigations: Targeted and non-targeted. Targeted strategy focuses on the isolation and quantification of a defined group of molecules and thus utilizes dedicated methodologies, whereas untargeted strategy enables obtaining global profile of molecules in a specimen, however, without quantitation data. Application of high-throughput, sensitive, and selective omics methodologies, mainly based on mass spectrometry, resulted in the more comprehensive characterization of venoms [19]. The use of state-of-the-art omics technologies has proven the pharmacological significance of HBV and enabled the optimization of therapeutic strategies by using selected, active components of HBV [20].

Venoms are complex mixtures of biologically active compounds including low-molecular-weight components like organic acids, nucleosides, amines, amino acids, and alkaloids. Analyses of various venoms and poisons indicated that some common constituents and also specific components occur in those secretions. Among unique components are acylpolyamines occurring in spider venoms, bufadienolides in toad poisons, and piperidine alkaloids in fire ant venoms, whereas monoamines and amino acids were found in many types of venomous and poisonous secretions [19]. The usefulness of the low-molecular-weight components in medicine was proved among other poisonous and venomous animals i.e., toads, frogs, snakes, and spiders [19]. However, the important active small molecules present in venoms and toxins are organic acids, which have not been fully explored yet [21].

In an attempt to better characterize HBV and understand its biological and pharmacological properties, we have performed analysis of organic acids in venom samples by using high-performance liquid chromatography-tandem mass spectrometry (HPLC-MS/MS). This is the first study presenting targeted analysis of this metabolite class in HBV and *Hymenoptera* venom in general. The research is mainly focused on organic acids involved in the citric acid cycle.

## 2. Results

### 2.1. Method Validation

A targeted metabolomic analysis was performed using HPLC-MS/MS system. Hydro-RP column and gradient elution were applied for chromatographic separation of organic acids. The mass spectrometer with triple quadrupole, which operated in the multiple reaction monitoring mode (MRM), was used as a detector. The optimized HPLC-MS/MS parameters were enabled to validate the method. Both sample preparation methods (dilute-and-shot and solid phase extraction (SPE)) were validated with good precision and accuracy. The calibration curves showed good linearity for all analyzed organic acids (r ≥ 0.993) (Table 1). The concentration ranges start from low ng/mL up to 1 µg/mL, except for citric acid (25 ng/mL–4 µg/mL) and fumaric acid (25 ng/mL–2.5 µg/mL). The LOQ was 5 ng/mL or 10 ng/mL for the majority of the analytes. The intra-batch precision and inter-batch precision were satisfactory and were ≤ 15.85% and ≤ 16.39%, respectively. The intra-batch accuracy varied between 81.71% and 113.50% and the inter-batch accuracy values ranged from 80.38% to 121.17%. All validation parameters of the method are listed in Table 1.

### 2.2. Organic Acid Profiles of HBV Samples

**Figure 1:**
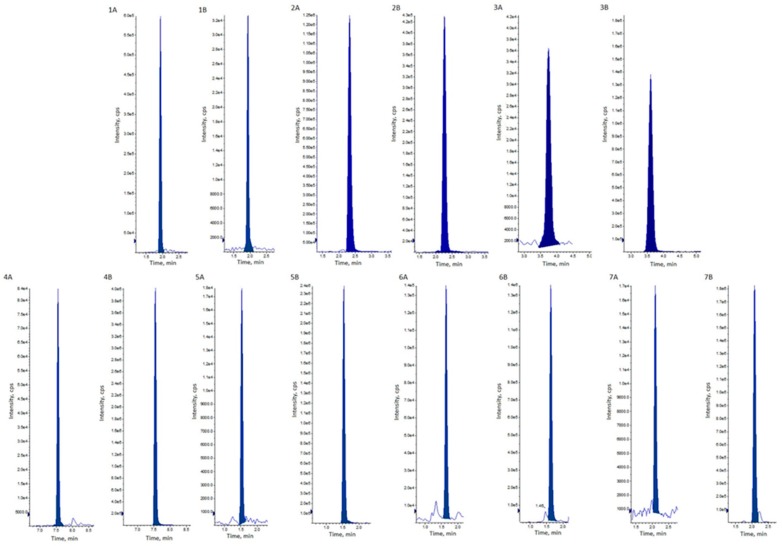
Extracted chromatograms of all analytes in honeybee venom samples (**A**) and standard solutions (**B**): 1—citric acid, 2—fumaric acid, 3—glutaric acid, 4—kynurenic acid, 5—malic acid, 6—malonic acid, 7—succinic acid.

The HPLC-MS/MS method enabled quantitative analysis of organic acids in HBV samples from 2018, 2017, and archival samples from 2006. All seven analyzed organic acids: Fumaric acid, citric acid, glutaric acid, kynurenic acid, malic acid, malonic acid, and succinic acid were present in each sample. Exemplary chromatograms are presented in Figure 1.

**Figure 2:**
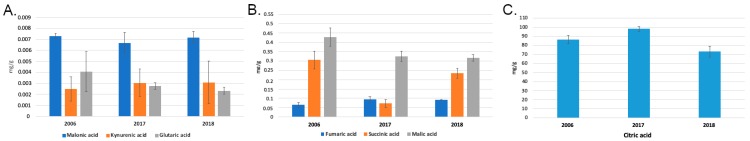
The concentration of organic acids in HBV samples from 2006, 2017 and 2018 (average ± SEM).

**Figure 3:**
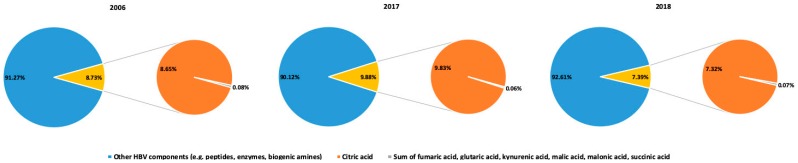
The percentage content of the analyzed organic acids in studied honeybee venom (HBV) samples (percentage of the dry weight of HBV). The dominant content of citric acid has been demonstrated.

Among the analytes, glutaric acid, kynurenic acid, and malonic acid were presented in HBV samples in the lowest concentrations. The content of these acids varied from 0.002 mg/g to 0.007 mg/g of the dry weight of venom (Figure 2A). In HBV collected in 2006, the kynurenic acid occurred in the lowest concentration level, whereas in the samples collected in 2017 and 2018, the glutaric acid was found in the lowest concentration. Despite that we observed variations in the concentrations of the studied organic acids between HBV, citric acid was the most abundant acid in each analyzed sample (Figure 2); it accounted for an average of 86 mg/g of the venom dry weight (Figure 2C). Due to huge amounts of citric acid and relatively high concentrations and good ionization of succinic acid and malic acid, the SPE sample preparation method was not optimal for the analysis of those organic acids, because we observed signal saturation. Therefore, we decided to prepare samples for the determination of these three acids by 2000-fold dilution. The sum of analyzed organic acids constituted from 7% to 10% of the dry weight of HBV and the determined organic acid profiles were clearly dominated by citric acid (Figure 3). We observed differences in the levels of individual organic acids in HBV between the years of sample collection. Among all analyzed organic acids, succinic acid (Figure 2B) showed the greatest variation of concentration between years (CV = 58.4%), whereas malonic acid was present in a similar concentration throughout all analyzed years (CV = 4.72%). The conducted measurement showed that the content of individual organic acid varied among years, however there is no trend in the changes. Surprisingly, the archival samples collected in 2006 did not show the lowest concentrations of the studied organic acids. This indicates that the applied storage conditions of HBV ensure satisfactory stability of the organic acids. Additionally, kynurenic acid showed the largest difference in concentrations within one year: In 2006, CV = 76.73%, in 2017, CV = 70.57%, and in 2018, CV = 108.32%. The concentrations of glutaric acid also varied significantly but only in 2006, where CV = 77.03%. Alternatively, concentrations of citric acid, malonic acid, and malic acid did not differ significantly in HBV samples collected within one year (Table 2).

## 3. Discussion

Venom metabolomics is a developing field of research, which provides an opportunity to explore new low-molecular-weight compounds by using modern approaches, such as mass spectrometry-based methodologies [19]. Venomics studies are still focused on the protein and peptide components of venoms and secretions [22,23,24]. Using the tag ‘*venom proteomics’* on the PubMed engine obtained 730 results while using *‘venom metabolomics’* obtained only 23 results. This proves that there is a need to expand the knowledge about the content and significance of small molecules occurring in animal venoms. In the current study, the application of the LC-MS/MS methodology enabled a quantitative analysis of seven organic acids in HBV samples. It was known that bee venom contains citric acid but there was a lack of information about other organic acids [25]. It was unknown which acids are present in HBV and in what quantities. Our research is the first study that examined the concentration of these metabolites in dry HBV using the liquid chromatography-mass spectrometry method, which provided specific and accurate data.

We determined the seven organic acids in samples from 2006, 2017, and 2018. The levels of individual organic acids were found to differ greatly both within a year and between years. The variations in the analytes’ HBV content collected in different years were also observed in the peptide analysis [26]. In the proteomics study, the same peptides were detected in HBV in a smaller amount in older samples (1991 and 1995) than in newer ones (2002 and 2007). These data demonstrate the effect of storage time on the peptide content in HBV through their degradation. However, we observed no similar trend in the determined profiles of organic acids. Surprisingly, the archival samples collected in 2006 did not show the lowest concentrations of each studied organic acid (Figure 1). For instance, in samples collected in 2006, we measured the lowest content of fumaric acid and the highest concentration of malic acid compared with samples from 2017 and 2018, whereas malonic acid was at a very similar level. This indicates that the applied storage conditions ensure the satisfactory stability of the organic acids in dry venom samples. We observed high variations in the levels of some acids between samples collected within the same year, which suggests biological causes of the observed differences in organic acid profiles. The obtained data cannot support the hypothesis on the effect of storage conditions on the concentrations of organic acids in venom. The variability of the examined organic acid levels could be due to many factors, such as habitat, food, age of bees, and weather conditions.

Organic acids are important components of animal venoms and secretions. Our study shows that the organic acid profile of HBV is dominated by citric acid (Figure 3). This acid is present in venoms of other species as well. Citric acid was identified as the most abundant component of snake venoms by gas liquid chromatography-mass spectrometry [27]. The study of 17 snake venoms (Elapidae, Viperinae, and Crotalinae) showed that citric acid was present in all venoms and was the first-ranked metabolite overall [25]. This acid was also found in spider venom [28,29]. Citric acid has chelating properties, and its presumed function is based on inactivation of metalloproteases, phospholipases, and nucleases to prevent the degradation of venom and glandular tissue. However, these enzymes are instantly reactivated after venom is injected into prey tissues [30]. Citrate inhibits the zinc ion dependent metalloprotease hemorrhagic toxins, and it is suggested that citrate has an inhibition activity on calcium ion dependent phospholipase A_2_ by binding Ca^2+^ [25]. The HBV contains phospholipase A_2,_ which is considered a major allergen in HBV for neurotoxic and myotoxic properties. Another supposed function of citric acid, which occurs in a high concentration in venom, is anticoagulation by inhibition of the coagulation cascade and platelet aggregation in the prey organism [21]. The anticoagulant activity may be relevant when introducing venom by sting or bite [31]. Citric acid plays a role as a buffer component and a negative counter ion for the basic peptides and acylopolyamines [29,31]. Moreover, citrate has antimicrobial activity in concentrations occurring in animal venoms [29].

The remaining organic acids (malic acid, succinic acid, fumaric acid, malonic acid, glutaric acid, and kynurenic acid) were detected and determined for the first time in HBV venom. Succinic acid and malic acid were previously found in snake venoms [21], whereas the presence of kynurenic acid was reported in toxin from frogs *Pipa carvalhoi* [32]. Venoms and toxins are similar secretions but differ in their way of application in the prey organism. Fumaric acid, malic acid, and succinic acid are intermediates in the citric acid cycle. Malonic acid is a competitive inhibitor against succinate dehydrogenase in the respiratory electron transport chain. This enzyme is responsible for the dehydrogenation of succinate in the citric acid cycle [33]. Glutaric acid is a product of the metabolism of amino acids, another important metabolite class found in HBV [4]. The 2-oxoglutaric acid is a derivative of glutaric acid, and it is a key molecule in the citric acid cycle [33]. Kynurenic acid properties include modifications of neuronal function by affect with receptors like NMDA receptors and neuronal cholinergic α7 nicotine receptors [34].

Our study demonstrates that low-molecular-weight compounds should not be neglected in venom research, but instead, they should be regarded as components worthy of investigation. In HBV samples, the analyzed organic acids constituted, on average, 8.7% of the dry weight of venom, which is in agreement with the study of Fenton et al. [25] where citric acid was determined by a coupled enzyme assay, aconitase-isocitric dehydrogenase. It is noteworthy that similar results were obtained by two completely different methods. The high content of organic acids lowers the pH of venom, this can be important in triggering pain in prey and in inhibition of bacterial growth [29]. It should be emphasized that citric acid constitutes 99% of the sum of organic acids determined in our study (Figure 3). Until now, sugars, biogenic amines and amino acids were listed as the main non-peptide components of HBV [4]. There are no organic acids on the list, however our study provided new evidence that HBV contains measurable levels of organic acids and confirmed that citric acid is a crucial bee venom component that should also be listed as an important non-peptide component of HBV. The developed method of the analysis of organic acids could also be adapted to study of other low-molecular-weight components in venom. However, there are some restrictions. A dilute-and-shot sample preparation method is suitable for metabolites on a similar concentration level as citric acid, malic acid and succinic acid in honeybee venom. In the SPE sample preparation method, the extraction columns appropriate for organic acids were used. Thus, they could be used for determination of acidic compounds.

Metabolomics approaches in venom studies can increase knowledge about venoms by investigating previously unexplored components. Thus, it is possible to understand the biological properties of venoms and improve treatment in the case of a bite or sting. However, metabolomics experiments are accompanied by difficulties and challenges. The main analytical challenge is the sample preparation method. The organic acids in HBV vary dramatically in a concentration ranging from between 0.002 mg/g for kynurenic acid to 86 mg/g for citric acid, i.e., it spans four orders of magnitude. Due to the significantly higher concentration of citric acid, the determination of other acids using one method of sample preparation was impossible. The key was to develop a method that allows not only such small compounds as organic acids to be identified, but also their determination. In our study, the solution was to use two sample preparation methods. Villar-Briones et al. also struggled with a similar challenge in a study of small organic compounds in snake venom [21]. The reported abundances of organic acids in snake venoms span nearly eight orders of magnitude. Therefore, heedful consideration of methods employed for sample preparation is extremely important in venom metabolomics as it can decide whether quantification of a given metabolite is feasible. Additionally, it is noteworthy that venom metabolome is a dynamic system susceptible to changes under the influence of an animal’s living environment, and may change rapidly. Therefore, the application of modern analytical techniques, such as LC-MS/MS, enables a better understanding of the pharmacological significance of biologically active molecules in animal venoms, thus opening the way to optimize therapeutic strategies.

The presented method has pros and cons. The simplicity of the methodology allows its use not only in venom studies, but also in analysis using other matrices. The selection of assayed organic acids was conscious. It is known that formic acid is present in animal venom, especially in ant venom [35]. However, it was not possible to determine formic acid in HBV samples because this acid was added to mobile phases as a modifier. The research was limited to the determination of citric acid, which is the most abundant organic acid in HBV, and acids associated with the citric acid cycle. Thus, the comprehensive organic acid profile of HBV has not yet been fully explored. The use of two protocols of sample preparation may seem laborious and troublesome, however, it is useful, especially when metabolite levels vary by several orders of magnitude. This approach enables quantitative analysis of metabolites over a wide concentration range.

## 4. Conclusions

This study expands our knowledge about the composition of HBV. It is the first study that provides specific and accurate data on the content of a panel of low-molecular-weight organic acids in HBV venom. The mass spectrometry-based methodology allows the determination of seven organic acids in HBV, with citric acid having the greatest abundance. In all analyzed HBV samples, citric acid constituted at least 7.3% of the venom dry weight. Future directions of HBV metabolomics include comparison organic acid composition of dry and fresh venom to find out the right concentration of these compounds during a sting. Addressing the question of what is the biological significance of organic acids in HBV requires further biological activities experiments to see the effects of excluding the organic acids from the HBV. Moreover, it is a future challenge to uncover the metabolic pathways and their mutual relations to understand the functioning of venoms, especially bee venom.

## 5. Materials and Methods

### 5.1. Reagents and Standards

All standards, including deuterated analogues used as internal standards (IS), were bought from Sigma Aldrich (St. Louis, MO, USA). The methyl-d_3_-malonic acid was used as IS for citric acid, fumaric acid, glutaric acid, malic acid, malonic acid, and succinic acid. The IS for kynurenic acid was kynurenic acid-d_3_. LC-MS grade methanol and formic acid were purchased from Sigma Aldrich (St. Louis, MO, USA). Deionized water (18.2 MΩ-cm resistivity at 25 °C) was obtained from Direct-Q3 UV water purifying system (Merck Millipore, Darmstadt, Germany).

### 5.2. Sample Collection and Preparation

The venom samples of *Apis mellifera* were collected in 2006, 2017, and 2018 from May to August (three samples for each year) from an apiary of the Department of Inorganic and Analytical Chemistry, Poznan University of Medical Sciences. HBV was obtained by electrical stimulation by using frames placed in the upper body of the hive in the middle space of the hive super. The samples were dried and stored until analysis at −80 °C.

At the moment of use, dried venom samples were dissolved in ultrapure water, vortexed (1 min), sonicated (15 min), and centrifuged (2 min, 10,000× *g*). The performed pilot study showed huge differences in the concentrations of the studied organic acids in HBV. Therefore, two different protocols for sample preparation were employed. For determination and validation of malonic acid, fumaric acid, glutaric acid, and kynurenic acid, the solid phase extraction was used. For solid phase extraction, Bakerbond SPE column processor (J.T. Baker, Griesheim, Germany) and Clean-up CUQAX extraction columns (200 mg, 3 mL, UCT, Bristol, TN, USA) were used. The amount of 50 µL of the obtained HBV solution (c = 50 mg/mL) was spiked with 5 µL of kynurenic acid-d_5_ as an internal standard (c = 2 µg/mL), then water was added to make 2 mL of the solution. For column conditioning, 3 mL of methanol and 3 mL of water were used. Subsequently, the prepared samples were applied, then the washing step was carried out with 3 mL of water and 3 mL of methanol. The analytes were eluted with 2 mL of methanol: Glacial acetic acid (94:6, v/v). Extracts were then evaporated at 35 °C (miVac Duo Concentrator, Genevac, Stone Ridge, NY, USA) and reconstituted with 100 µL of solvent A (0.2% formic acid in water). The second method was optimal for quantification of acids occurring in HBV in high concentration: Citric acid, malic acid, and succinic acids. The samples were prepared by mixing 5 µL of HBV solution (c = 50 mg/mL) with 10 µL of methyl-d_3_-malonic acid as an internal standard (c = 100 µg/mL) and adding the mobile phase A to 10 mL (total dilution factor of 2000). After vortexing and centrifuging, the supernatants were subjected to HPLC-MS/MS analysis.

### 5.3. Liquid Chromatography

Chromatographic separation was performed using 1260 Infinity high-performance liquid chromatograph (Agilent Technologies, Santa Clara, CA, USA). Analytes were eluted from a Synergi Hydro-RP column (4 µm, 150 mm × 2.0 mm, 80Å, Phenomenex, Torrance, CA, USA) at a flow rate of 300 µL/min at 50 °C. The gradient elution of solvent A (0.2% formic acid in water) and solvent B (0.2% formic acid in methanol) was programmed as follows: 0–2 min with 97% solvent A, 2 min–4.5 min linear from 97% to 3% solvent A, 4.5 min–5 min with 3% solvent A, 5 min–5.5 min from 3% to 97% solvent A, 5.5 min–10 min with 97% solvent A. Injection volume was constant and equal to 10 µL.

### 5.4. Mass Spectrometry

Detection was performed on a 4000 QTRAP mass spectrometer (Sciex, Framingham, MA, USA) and controlled with Analyst Software version 1.5.2 (Sciex, Framingham, MA, USA). The mass spectrometer was equipped with an electrospray ionization TurboV ion source, which operated in negative ion mode. The source parameters were as follows: Ion spray voltage, 4.5 kV; gas source 1, 40 psig; gas source 2, 50 psig, and ion source temperature, 600 °C. Multiple reaction monitoring (MRM) mode with two transitions for each analyte was used. The optimized LC-MS/MS parameters used in MRM acquisition mode are shown in Table 3. The most abundant transitions allowed quantification, and the second transitions were used for identity confirmation of each compound.

### 5.5. Method Validation

To achieve the highest level of selectivity, the MRM mode with two MRM transitions for each analyte was used. Linearity of the method was examined by using internal standard calibration. The calibration curves were constructed using linear regression, and were prepared in various ranges to cover different concentration levels of analytes observed in HBV samples (Table 1). The lowest standard concentration on the calibration curve was regarded as the limit of quantification (LOQ) and was determined on the basis of a signal-to-noise (S/N) ratio of 10 and acceptable values of accuracy (bias ≤ 20%) and precision (CV ≤ 20%). Precision and accuracy were calculated by analyses of the pooled HBV sample spiked with the organic acids (quality control samples, QC) at three different levels (low, medium, and high). To estimate intra-batch precision, each QC sample was prepared and analyzed five times in a single run. The inter-batch precision was determined by analyzing five replicates of each QC sample in three different runs. Accuracy was calculated as (spiked sample result-unspiked result)/known spike added concentration × 100%.

## Figures and Tables

**Table 1:** Validation parameters for HPLC-MS/MS method.

Analyte	Concentration Level (ng/mL)	Accuracy		Precision		Method Calibration Range (ng/mL)	Linearity (r)	LOQ (ng/mL)
		Intra-batch (RSD, %)	Inter-batch (RSD, %)	Intra-batch (RSD, %)	Inter-batch (RSD, %)			
Citric acid	200	99.17	87.92	6.82	15.36	25–4000	0.9943	25
	750	96.22	89.33	7.50	12.85			
	1500	104.78	111.98	14.63	15.61			
Fumaric acid	50	92.80	107.30	2.96	10.25	25–2500	0.9947	25
	200	113.50	101.75	9.23	3.30			
	750	93.39	82.05	9.16	9.00			
Glutaric acid	50	91.00	116.50	11.21	11.95	10–1000	0.9990	10
	200	87.25	100.50	2.07	12.93			
	750	87.67	93.50	8.98	6.74			
Kynurenic acid	50	92.56	91.71	15.13	12.52	5–1000	0.9993	5
	200	92.64	102.39	9.48	10.59			
	750	88.30	101.07	4.87	11.59			
Malic acid	100	100.80	121.17	3.45	16.39	5–1000	0.9987	5
	200	99.60	105.50	9.19	12.35			
	750	94.51	107.20	7.32	14.82			
Malonic acid	50	86.00	108.00	4.79	18.76	5–1000	0.9926	5
	200	88.50	89.50	0.63	12.59			
	750	81.71	80.38	1.69	12.19			
Succinic acid	100	87.30	86.70	2.99	2.91	5–1000	0.9969	5
	200	95.32	94.96	2.52	14.19			
	750	100.35	93.29	1.95	8.89			

**Table 2:** Average values of determined organic acids along with standard deviations (SD) and relative standard deviations (RSD) in samples from 2006, 2017, and 2018.

Organic Acid	2006			2017			2018		
	AVERAGE (mg/g)	SD (mg/g)	RSD(%)	AVERAGE (mg/g)	SD (mg/g)	RSD(%)	AVERAGE (mg/g)	SD (mg/g)	RSD(%)
Citric acid	86.5333	8.3363	9.6336	98.2667	4.8881	4.9743	73.2000	10.5357	14.3930
Fumaric acid	0.0643	0.0222	34.4324	0.0937	0.0255	27.2133	0.0904	0.0053	5.8660
Glutaric acid	0.0041	0.0031	77.0317	0.0028	0.0005	17.1915	0.0023	0.0005	22.4465
Kynurenic acid	0.0025	0.0019	76.7334	0.0030	0.0022	70.5674	0.0031	0.0034	108.3253
Malic acid	0.4287	0.0858	20.0258	0.3246	0.0481	14.8040	0.3170	0.0295	9.3062
Malonic acid	0.0073	0.0004	5.5610	0.0067	0.0017	25.0431	0.0072	0.0009	13.0087
Succinic acid	0.3051	0.0826	27.0575	0.0726	0.0364	50.0559	0.2339	0.0468	20.0149

**Table 3:** HPLC-MS parameters optimized for the multiple reaction monitoring (MRM) mode.

Compound	Molecular Weight (Da)	Retention Time (min)	Precursor Ion (m/z)	Product Ion (m/z)	DP (V)	EP (V)	CE (V)	CXP (V)
Citric acid	192.12	1.92	190.9	86.9	−53	−10	−24	−24
Fumaric acid	116.07	2.28	115.0	71.0	−42	−10	−12	−12
Glutaric acid	132.11	3.69	131.0	86.8	−40	−10	−17	−17
Kynurenic acid	189.17	7.56	187.9	144.0	−45	−10	−23	−23
Malic acid	134.09	1.52	132.9	115.0	−40	−6	−16	−16
Malonic acid	104.06	1.65	102.9	59.0	−35	−12	−14	−14
Succinic acid	118.09	2.17	117.0	73.0	−45	−9	−18	−18
Kynurenic acid-d_5_*	194.20	7.46	192.9	148.9	−55	−7	−20	−13
Methyl-d_3_-malonic acid*	121.11	2.82	119.9	76	−33	−10	−15	−12

DP: Declustering potential; EP: Entrance potential; CE: Collision energy; CXP: Collision cell exit potential; *internal standard.
